# Supplementary material for: Investigation of Plasma-Derived Lipidome Profiles in Experimental Cerebral Malaria in a Mouse Model Study
Source: Int J Mol Sci. 2022 Dec 28;24(1):501. doi: 10.3390/ijms24010501 (PMC9820457; doi:10.3390/ijms24010501)
Supplement: Supplementary file 1 [file ijms-24-00501-s001.zip › ijms-1938648-supplementary.pdf]

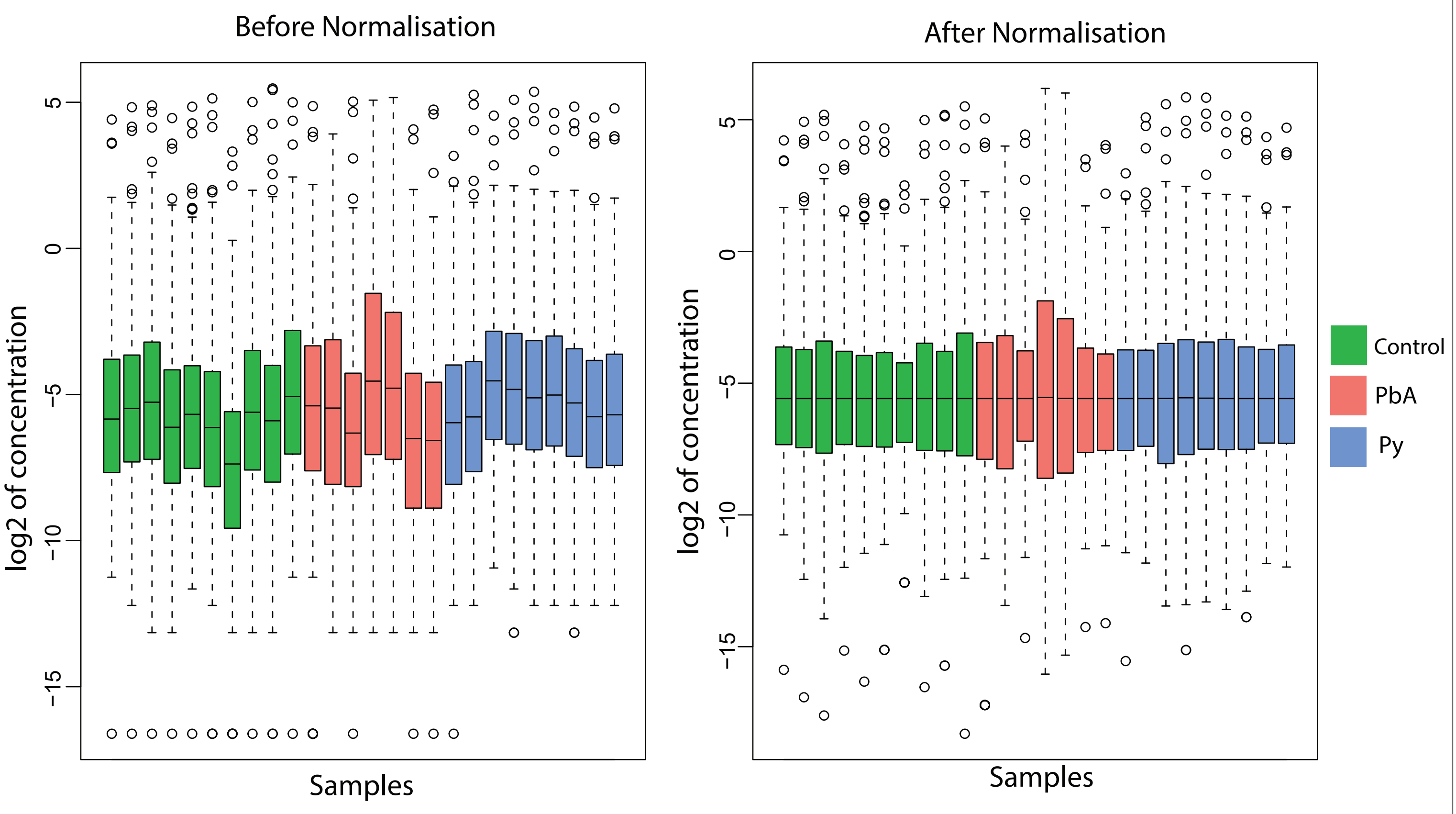

Supplementary Figure S1. **Boxplots of samples before and after median normalisation.** Each graph represents the minimum, maximum, median, first quartile and third quartile in the data set. Circles are outliers. An outlier is defined as a data point that is located outside 1.5 times the interquartile range above the upper quartile and bellow the lower quartile

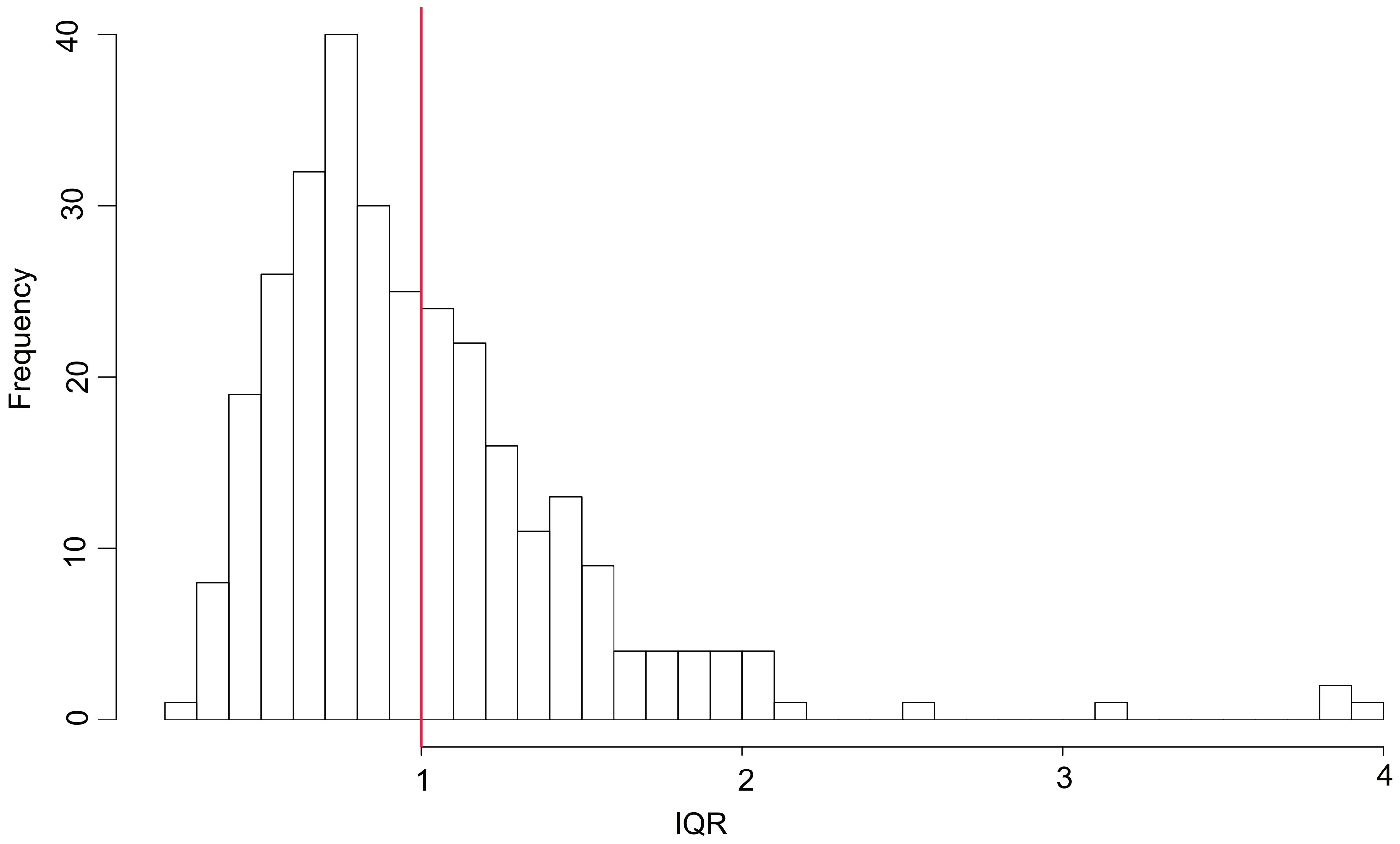

Supplementary Figure S2. Histogram of Interquartile range (IQR) representing variability of lipids across samples. Lipids with IQR ≤ 1 (red line) were considered as invariant and removed for further analysis
